# Investigating the Potential Use of Chemical Biopsy Devices to Characterize Brain Tumor Lipidomes

**DOI:** 10.3390/ijms23073518

**Published:** 2022-03-24

**Authors:** Joanna Bogusiewicz, Bogumiła Kupcewicz, Paulina Zofia Goryńska, Karol Jaroch, Krzysztof Goryński, Marcin Birski, Jacek Furtak, Dariusz Paczkowski, Marek Harat, Barbara Bojko

**Affiliations:** 1 Department of Pharmacodynamics and Molecular Pharmacology, Faculty of Pharmacy, Collegium Medicum in Bydgoszcz, Nicolaus Copernicus University in Torun, 85-089 Bydgoszcz, Poland; j.bogusiewicz@cm.umk.pl (J.B.); gorynska@cm.umk.pl (P.Z.G.); karol.jaroch@cm.umk.pl (K.J.); gorynski@cm.umk.pl (K.G.); 2 Department of Inorganic and Analytical Chemistry, Faculty of Pharmacy, Collegium Medicum in Bydgoszcz, Nicolaus Copernicus University in Torun, 85-089 Bydgoszcz, Poland; kupcewicz@cm.umk.pl; 3 Department of Neurosurgery, 10th Military Research Hospital and Polyclinic, 85-681 Bydgoszcz, Poland; mbirski@poczta.fm (M.B.); jacek.furtak2019@gmail.com (J.F.); darek_paczkowski@vp.pl (D.P.); 4 Department of Neurosurgery and Neurology, Faculty of Health Sciences, Collegium Medicum in Bydgoszcz, Nicolaus Copernicus University in Torun, 85-168 Bydgoszcz, Poland

**Keywords:** brain tumor, glioma, SPME, heterogeneity, lipids, chemical biopsy

## Abstract

The development of a fast and accurate intraoperative method that enables the differentiation and stratification of cancerous lesions is still a challenging problem in laboratory medicine. Therefore, it is important to find and optimize a simple and effective analytical method of enabling the selection of distinctive metabolites. This study aims to assess the usefulness of solid-phase microextraction (SPME) probes as a sampling method for the lipidomic analysis of brain tumors. To this end, SPME was applied to sample brain tumors immediately after excision, followed by lipidomic analysis via liquid chromatography-high resolution mass spectrometry (LC-HRMS). The results showed that long fibers were a good option for extracting analytes from an entire lesion to obtain an average lipidomic profile. Moreover, significant differences between tumors of different histological origin were observed. In-depth investigation of the glioma samples revealed that malignancy grade and isocitrate dehydrogenase (IDH) mutation status impact the lipidomic composition of the tumor, whereas 1p/19q co-deletion did not appear to alter the lipid profile. This first on-site lipidomic analysis of intact tumors proved that chemical biopsy with SPME is a promising tool for the simple and fast extraction of lipid markers in neurooncology.

## 1. Introduction

Malignant primary brain tumors are one of the most life-threating neoplasms. The majority of these tumors are classified as gliomas, which are divided into many types and subtypes based on their histological features and the results of genetic testing (e.g., mutations in gene coding isocitrate dehydrogenase or co-deletion of the 1p/19q chromosome) [1]. The early detection of gliomas is challenging due to their non-specific symptoms and tendency to manifest later in their development. In most cases, the excision of these neoplastic lesions with appropriate margins is essential to improving the patient’s outcome. This approach is generally followed by the application of chemo- and/or radiotherapy as a complementary treatment method. A patient’s survival prognosis varies depending on the tumor subtype, with prognoses as short as a few months for patients diagnosed with glioblastoma [2,3,4]. The poor prognoses and high recurrence rates for gliomas are directly related to the lack of effective treatment methods. Moreover, the locations of brain lesions often make them difficult to access, which is another key obstacle in the development of new sampling approaches. As such, it is important to explore new opportunities offered by translational research that link medicine and analytical chemistry [3]. Thus, the application of “omics sciences” such as metabolomics, lipidomics, and genomics could provide a useful tool for determining biomarkers that could help with the selection and monitoring of treatments.

Lipidomics is a branch of metabolomics that focuses on lipid species, and findings have shown that it can play an important role in the biomarker search workflow. Lipids perform various functions in organisms, which can change as a result of carcinogenesis [5,6]. These alterations can be analyzed using many different analytical platforms, with the majority being based on mass-spectrometry detection and identification [7,8]. It is important to note that solid samples such as tissues must be properly prepared prior to instrumental analysis, which generally entails homogenization and analyte extraction. Not only are these protocols time consuming and labor intensive, but they also preclude the use of the sample for additional tests, as it has been permanently modified [9]. To avoid tissue disintegration, researchers have worked to develop new approaches that do not require sample homogenization, such as ambient mass-spectrometry methods (e.g., desorption electrospray ionization mass spectrometry (DESI-MS), matrix-assisted laser desorption/ionization mass spectrometry (MALDI-MS)) [10]. However, such analytical methods still require tissue to be excised and cut into thin slices. In contrast, solid-phase microextraction (SPME) enables tissue analysis without any pre-treatment, which has led to its use in a wide range of areas, including neuroscience, pharmacotherapy, and organ transplantation [11,12,13,14,15,16,17,18,19,20,21].

SPME is based on the interaction between analytes and a sorbent coating, which extracts the targeted molecules until equilibrium is reached. However, in the case of untargeted clinical studies, optimizing the extraction time is the best compromise between clinical practicality and extraction efficiency for the metabolites of interest. Furthermore, SPME extraction does not require tissue consumption, as only small molecules are withdrawn from the studied sample; hence, SPME is sometimes also referred to as “chemical biopsy” [21]. Although a number of SPME geometries have been developed to date, including meshes, membranes, and blades [11], coated fibers remain the most popular. SPME fibers consist of a core made from a nickel-titanium alloy rod, and one end coated with an adequate sorbent (e.g., hydrophilic-lipophilic balanced (HLB) or octadecyl (C18)). The diameter of the SPME probe is approximately 200 μm, which makes it thinner than a classical brain biopsy needle (ca. 1.8 mm) and enables the sampling of analytes without significantly disrupting the studied lesion or organ [11,12,20,22]. Apart from minimal invasiveness during sampling, SPME fibers offer a few other key features that are useful in tissue analysis. Firstly, chemical biopsy is a non-depletive method, which means that only a negligible portion of the free fraction of analytes is sampled under given conditions. As a result, metabolic pathways are not interrupted during sampling, which allows sampling to be repeated multiple times to obtain temporal data, particularly in the case of in vivo studies, as the biological system is able to immediately recover (re-balance) [21]. Secondly, sampling via SPME facilitates the quenching of the metabolic reaction, as the sorbent only permits the extraction of small molecules, which enables the analysis of even unstable substances [23]. Moreover, the chemical biopsy protocol is simple and fast. During sampling, the probe is simply inserted into the tissue for a predetermined amount of time and then rinsed with water to remove potential remnants of tissue or biological fluids that may be loosely attached. The probe can then be transported to a lab and desorbed immediately or stored until instrumental analysis [11,12].

SPME probes have been previously employed in metabolomic analyses of brain tumors, but their use in in-depth lipidomic profiling has yet to be investigated [13,24]. Therefore, the aim of the present study is to assess the utility of chemical biopsy in the lipidomic analysis of brain tumors. To this end, a proof-of-concept experiment was first carried out to evaluate the utility of SPME probes in differentiating tumors with different histological origins (meningiomas and gliomas). This was followed by a detailed analysis of gliomas, which entailed using SPME to assess the intra- and inter-tumor distribution of lipids in neoplastic lesions The lipidomic profile of these lesions was also assessed with respect to malignancy grade, isocitrate dehydrogenase mutation status, and the presence of 1p/19q codeletion.

## 2. Results

### 2.1. Assessing Tumor Heterogeneity Based on Lipidome Composition

**Figure 1:**
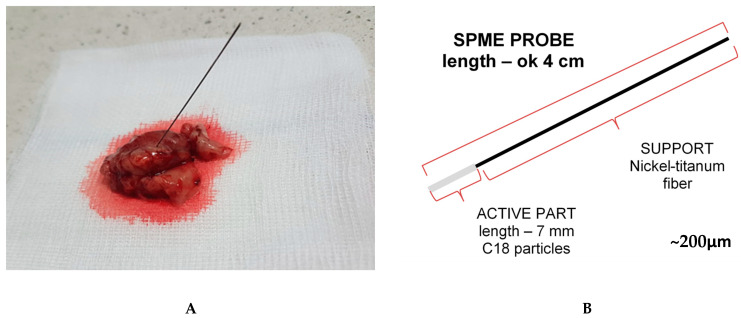
Chemical biopsy probes: (**A**) SPME probe during the sampling of a brain tumor; (**B**) the construction of the SPME probes.

The reliability of SPME probes for the analysis of heterogeneous tumors was assessed by using three 7 mm C18 probes (Figure 1) to perform extractions from four meningiomas and four high-grade gliomas, followed by lipidomic analysis.

**Figure 2:**
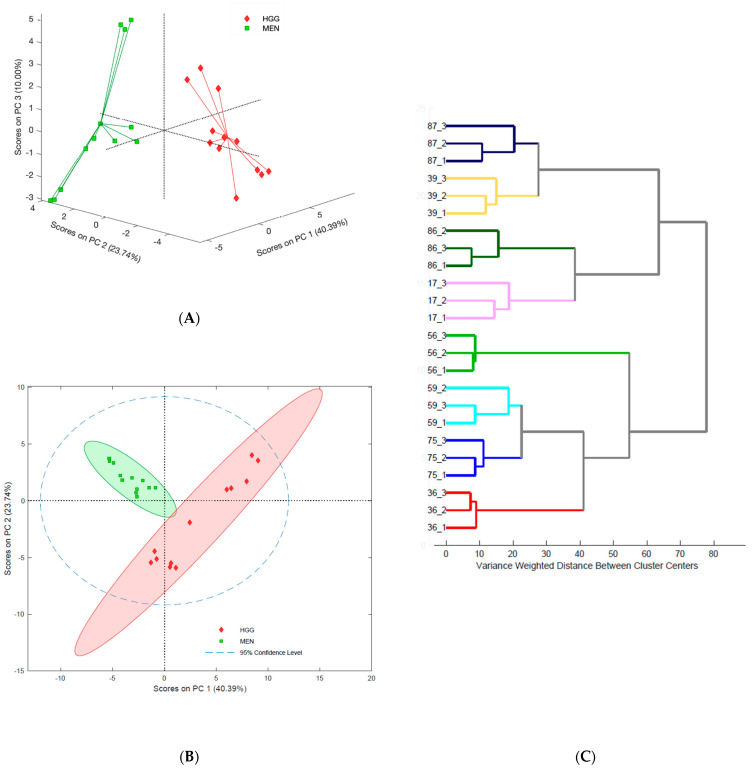
(**A**) Three-dimensional principal component analysis plot; preprocessing of variables: log10 and autoscale. (**B**) Two-dimensional principal component analysis plot showing samples from the same tumor; preprocessing of variables: log10 and autoscale. (**C**) Hierarchical clustering dendrogram based on tentative lipids with a VIP score above 1.0; preprocessing of variables: log10 and autoscale. Samples 36, 56, 59, and 75 were meningiomas, and samples 17, 39, 86, and 87 were gliomas. The number after the lower dash denotes the replicate inserted into the same lesion.

It was observed that these histologically different tumors formed two distinct and separate groups on a three-dimensional PCA plot built on all tentatively detected lipids (Figure 2A). Nonetheless, diversity in each group was observed. A subsequent two-dimensional PCA plot showed that samples obtained from the same tumor type were situated close to each other (Figure 2B). Furthermore, a high degree of correlation was observed in lipidome data obtained from the same tumor with all three probes. The correlation plots for these data were presented in Supplementary Materials (Figures S1 and S2). In addition, cluster analysis showed that the samples formed two groups that further divided into subgroups corresponding to individual patients, indicating that intra-tumor variability was significantly smaller than inter-tumor differences (Figure 2C). A quick review of the variation in the results was then performed by examining the coefficients of variation for lipid species with structures confirmed by MS/MS analysis (Table S2). We observed that, in the majority of cases, the coefficient of variation (CV) for a particular tumor was below 30%. The presence of HexCer was characterized by higher CV values and lower normalized peak areas. Significantly higher variation was observed when calculating the average CV value of a given lipid in a given tumor type, which was driven by inter-patient variability (Table S1).

### 2.2. Differentiation of Brain Tumors

Following the heterogeneity experiment, the lipidomic profiling of meningiomas and gliomas was conducted using a bigger cohort. In addition, a more in-depth analysis of gliomas that assessed their grade, IDH mutation status, and 1p/19q co-deletion status was conducted. The proposed method enabled the extraction of a wide range of tentative lipids, as well as the structural confirmation of 15 phosphatidylcholines (PC), 46 phosphatidylethanolamines (PE), 10 lysophosphatidylcholines (LPC), one phosphatidylethanolamine (LPE), 13 sphingomyelins (SM), 5 ceramides (Cer), 18 hexoylceramides (HexCer), 2 lactosylceramides (LacCer), and one sulfatide (ST d42:2) via MS/MS analysis. A list of identified lipid species is provided in Table S2. Lipid species with MS/MS-confirmed structures received greater attention in further analyses than those that were only identified tentatively.

#### 2.2.1. Lipidomic Profiles of Brain Tumors with Different Histological Origins

The lipidomes of both types of tumors largely consisted of phospholipids, with PEs as one of the major groups at approximately 49% and 46% for meningiomas and gliomas, respectively (Figure S3A,B). PCs comprised the second largest group of lipids at 27% for meningiomas and 24% for gliomas. The remaining lipid groups were made up of lyso- forms of PCs and PEs, as well as sphingolipids. More in-depth analysis revealed that gliomas had significantly higher levels of Cer and HexCer compared to meningiomas and that both tumors had coefficients of variation that were higher than 100%. Gliomas also contained increased levels of sphingolipids and phospholipids, but the CVs for these lipids was lower than 50%. Notably, LPE and ST should not be analyzed separately, as only one species of each group was identified in this experiment: LPE P-16:0 and ST 42:2;O2, respectively. A detailed account of the comparative statistical analysis results is provided in the Supplementary Materials (Table S3).

**Figure 3:**
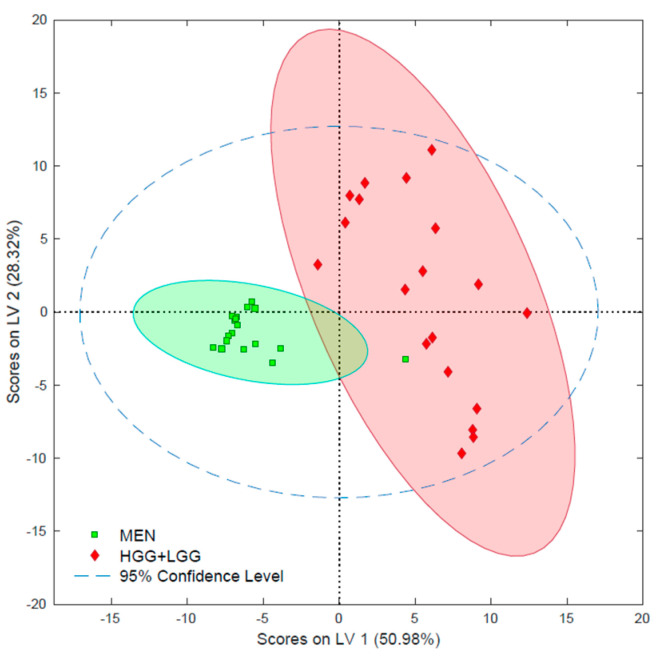
Chemometric analysis of meningiomas and gliomas—partial least squares data analysis (PLS-DA) performed on all detected features with a VIP above 1.0.

Chemometric analysis of tentatively identified lipids with a VIP-score above 1.0 confirmed the good separation of meningiomas and gliomas that had been observed previously (Figure 2 and Figure 3). This analysis was confirmed through a positive permutation test and acceptable errors. All of the parameters used in the chemometric analysis (Table S4) and the PCA (Figure S4) are presented in the Supplementary Materials.

#### 2.2.2. Lipidomic Differentiation of Gliomas with Different Grades

**Figure 4:**
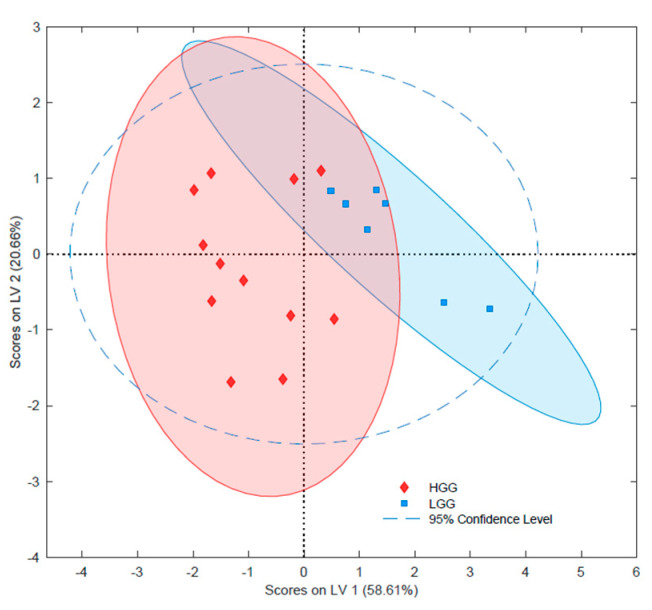
Principal least squares data analysis (PLS-DA) of gliomas with different grades. HGG—high grade glioma; LGG—low grade glioma.

Tumor grade was not associated with significant variability in the lipidome. Detailed statistical analysis of tentatively identified lipids revealed significant changes in 17 species between high-grade gliomas (HGG) and low-grade gliomas (LGG) (Table S5). It should be noted that the set of lysophospholipids with the highest alterations (i.e., LPC P-16:0, LPC P-18:0, LPC 18:2) were present in HGG at over twice the level as in LGG. On the other hand, among lipids with confirmed structures, lower concentrations of one SM were observed in HGG. Nevertheless, based on the selection from all significantly altered tentative lipids, a pattern of only four analytes enabled the differentiation of low-grade gliomas and high-grade gliomas in the PLS-DA model with satisfactory parameters (Figure 4 and Table S4). Among those lipids was LPC P-16:0, which was present in HGG at twice the concentration it was in LGG, and SM 42:1;O2, which was present at lower levels in higher-grade samples. The PCA can be found in the Supplementary Materials (Figure S5).

#### 2.2.3. Lipidomic Differentiation of Gliomas with Various IDH1/2 Mutation Statuses

**Figure 5:**
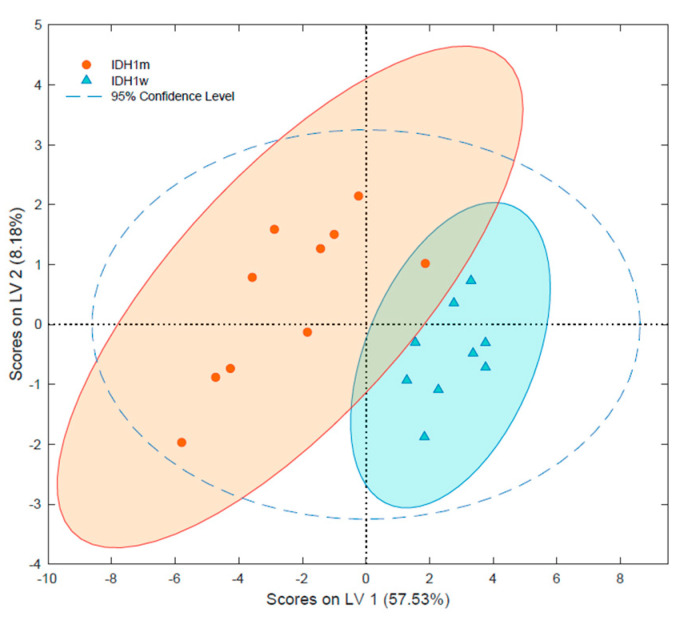
Partial least square data analysis (PLS-DA) of gliomas with different IDH mutation statuses. IDHm—isocitrate dehydrogenase gene mutant; IDHw—isocitrate dehydrogenase gene wiltype.

IDH 1/2 mutation status is considered to be one of the most important factors in the clinical diagnosis of gliomas and a good prognosticator of survival [25]. Therefore, an attempt was made to profile the lipidomes of gliomas with and without IDH mutation. The results showed that wildtype tumors (IDHw) had significantly increased LPCs (i.e., LPC O-16:0; LPC P-18:0; LPC 18:0) and lower levels of PCs such as PC 32:1, PC 32:0, and PC 36:1 (Table S7). Notably, wildtype tumors also contained decreased levels of PEs with confirmed structures. While sphingolipids levels generally remained unaltered, two of them, SM 43:4;O2 and LacCer 42:2;O2, were present at increased levels in the wildtype samples. Moreover, it was possible to select the set of 16 tentative lipid species that enable differentiation of gliomas based on the IDH 1/2 mutation status (Figure 5 and Table S4). Among these analytes were the phosphatydyloethanoloamines PE O-36:4, PE 38:4, and PE O-40:6, which were present at higher levels in the mutant samples. In contrast, LPC O-16:0 and SM 43:4;O2 were downregulated in the mutant samples (IDHm). The complete set of differentiating tentative lipids (Table S6) and the PCA plot can be found in the Supplementary Materials (Figure S6).

#### 2.2.4. Lipidomic Differentiation of Gliomas with Various 1p/19q Co-deletion Statuses

**Figure 6:**
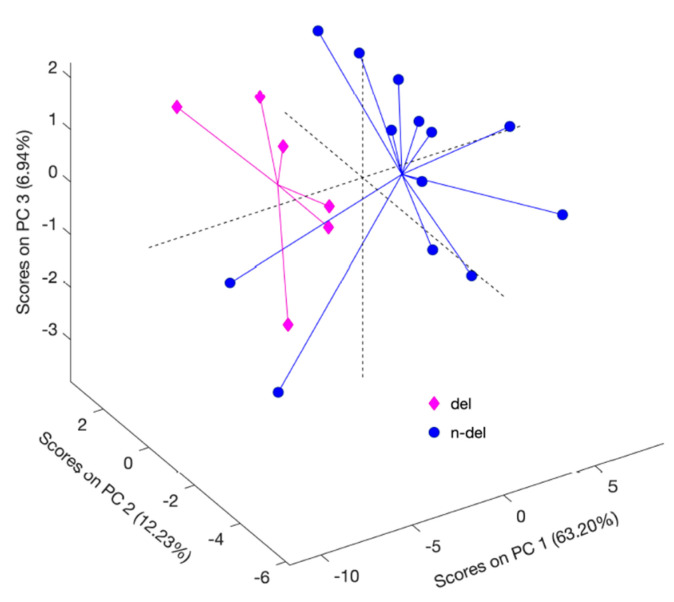
Three-dimensional principal component analysis of brain tumors with different 1p/19q co-deletion statuses. Autoscaling and logarithmic transformation were applied; del- 1p/19q co-deleted samples; n-del- 1p/19q non co-delated samples.

The lipidomes of neoplasms with different 1p/19q codeletion statuses were characterized by similar lipid composition. Detailed analysis of all of the tentatively identified lipids revealed that 36 were significantly altered in these two types of tumors (Table S7). Specifically, the lysophospholipids LPC O-16:0, LPC P-16:0, LPC 18:0, and LPC P-18:0 were present at higher levels in the non-co-deleted samples, while fully acetylated phospholipids were mainly present at lower levels (Table S7). However, due to the weak parameters of the model, it was not possible to select a set of lipids that could be used to discriminate mutants from wildtype co-deleted and non-co-deleted samples with statistical significance (Figure 6).

## 3. Discussion

The biochemical analysis of cancerous tissue can be challenging; therefore, new approaches capable of simplifying this procedure and expanding the range of obtained information are required. One such potential method is chemical biopsy, which combines sampling and sample preparation into one step, making it easy to use in clinical applications. SPME probes have been previously applied to perform extractions from cell lines and animal and human samples in biomedical studies aimed at examining various medical procedures and diseases (e.g., therapeutic drug monitoring, organ transplantation, malignant hyperthermia) [11,14,16,19,26]. However, only a few reports exist relating to the use of SPME in cancer research. For instance, Goryńska et al. [13] were able to apply SPME to select a distinctive set of metabolites to describe tumors with different histological origins, grades, and genetic mutations. In addition, other findings have shown that the acylcarnitine profile of gliomas changes based on the molecular and histological features of a tumor, which could be related to changes in lipid composition [24]. Lipidome analysis via SPME probes has also been investigated from an analytical perspective, as well as for its usefulness in biomedical applications [18,27,28,29]. The results of the above studies provide a solid foundation for the assessment of brain tumor lipidomes using chemical biopsy. Cancerous cells are characterized by their high proliferation rate and metabolic plasticity [30,31]. These features enable them to adapt well to changing conditions, which contributes to the development of a wide range of changes in their structure and functions. These alterations result in diversity at the inter-tumoral (between patients) and intra-tumoral (heterogeneity within the same lesion) levels [32]. As such, the selection of a set of biomarkers that can enable the stratification of tumors with satisfactory specificity, in addition to providing accurate and fast assessment of patient outcomes, is highly challenging [33]. To address this challenge, we analyzed how tumor heterogeneity—specifically, heterogeneity within and between meningiomas and gliomas—impacts the reliability of lipidomic profiling via chemical biopsy. As expected, unsupervised analysis revealed clear separation between the different histological types of brain tumor (Figure 2). Although inter-patient variability was observed in both groups, the group containing glioma samples demonstrated higher variability defined by PC2 (Figure 2A,B). In general, the inter-patient variability can be explained by diversity among the patients with respect to the location of the tumor, age, sex, lifestyle, and other environmental factors; nonetheless, the characteristics of the glioma samples featured a much higher degree of molecular alteration overall.

Intra-tumoral diversity is an additional challenge associated with the use of SPME probes, as these devices only extract analytes from a small area around the fiber. Various approaches can be used to characterize the spatial heterogeneity of tumors on a histological, genetic, transcriptomic, epigenetic, and metabolomic level [32,34,35]. Thus, there is significant risk of obtaining false results due to sampling only a small area. However, the inter-probe reproducibility results associated with the sampling of a given tumor showed high correlation factors between the lipid profiles obtained by individual probes (Figures S1 and S2). Ultimately, the data show that the set of extracted lipids for all fibers will be characteristic of that particular lesion, and that eventual variances will be relatively insignificant. As can be seen in the cluster analysis shown in Figure 2C, the samples from specific tumors are closer to each other than they are to samples obtained from different patients, despite being the same type of tumor (Figure 2C). Therefore, the diversity among the samples can be largely explained by the diversity of the patients. Variations in the level of analytes in the same tumor were acceptable in most cases, with the exception of ceramides and their derivates; indeed, low levels of these lipids in the studied samples may explain the high CVs that were obtained (Table S1). Another possible explanation could be related to decreased levels of some lipids due to the partial degradation of samples during storage between excision and their use for extractions [36]. Nevertheless, based on the current findings, it can be concluded that long fibers are a good option for extracting analytes from an entire lesion to obtain an average lipidomic profile. Alternatively, chemical biopsy tools such as miniaturized SPME probes enable dot-sampling, which was developed by Vasiljevic et al. [37], and can be employed to sample small target areas, such as profiling of necrotic lesion or calcification region. Another tool that has been applied to obtain spatially resolved data is an SPME device consisting of four fibers, which was successfully deployed to extract analytes from grey and white matter during neurosurgical procedures [21]. In addition, the ability of a single fiber to capture the average lipidome composition of an entire lesion is valuable with respect to the future use of this technology in in vivo studies. At present, there are numerous reports documenting the use of SPME probes on human subjects during surgical procedures or biopsies [14,21]. The ability to insert a chemical biopsy probe into a pathologically changed lesion followed by fast detection (e.g., with microfluid open interface mass spectrometry (MOI-MS) or coated-blade-spray mass spectrometry (CBS-MS)) indicates this approach’s immense potential for use in intraoperative diagnostics [38].

Next, investigations were conducted on larger group of meningiomas and gliomas with more complex characteristics (different grades and mutations) (Table S8). Our results detected two separate groups of meningiomas and gliomas (from 2nd to 4th grade), which was consistent with the findings of other researchers who analyzed the metabolic profile of these types of lesions using different platforms, such as DESI-MS and magnetic resonance imaging (MRI) [13,39]. HILIC chromatography, which was employed to separate hydrophilic lipids based on their representative polar head-group classes, enabled the detection of a wide range of phospholipids and sphingolipids (Table S2) [7,40]. Interestingly, the results revealed that gliomas, which are higher malignancy neoplasms compared to meningiomas, contained higher amounts of ceramides and their derivates (i.e., HexCer), while meningiomas contained higher levels of SMs, PCs, PEs, and LPCs (Figure S3 and Table S3). These alterations did not align with previous reports on biochemical alterations in gliomas compared to healthy tissue [6,41,42]; thus, further studies, possibly including spatially resolved in vivo sampling of healthy and cancerous tissue, are required to investigate this discrepancy in greater depth. It must also be emphasized that the above-described comparison was performed as a proof-of-concept to verify the proposed method’s applicability for the differentiation of brain tumors, and that further in-depth biological interpretation and discussion of these results was not undertaken, as currently available clinical tools already enable the easy identification of these lesions [25]. Given the relatively easy diagnosis of meningiomas compared to the high complexity resulting from the histological and molecular diversity in gliomas, further studies using chemical biopsy to examine the lipidomic profiles of gliomas were performed to assess this method’s viability for future use in the diagnostic process.

The ability to accurately assess the grade of a malignant tumor is critical in the prognosis of a patient’s survival. However, histological examination is often difficult due to the high diversity of glioma cells and the microenvironment of the tumor [32,35,43]. Examinations of a tumor via microscope are subjective and dependent on the pathologist’s experience, which can result in misclassification [43]. The metabolomic or lipidomic phenotype of a tumor reflects not only its histopathological information, but also contains all of the information about the given sample. As such, it is possible to select the biochemical features corresponding to the given characteristics of the sample (e.g., malignancy grade) by comparing the metabolomic/lipidomic data with a reference. In the current study, the PLS-DA comparing the lipidomic profiles obtained for gliomas and the histology results for their malignancy grades shows two separate groups corresponding to LGG and HGG (Figure 4 and Table S4). However, some slight overlapping was observed among some samples, which could indicate that malignant neoplasms undergo dynamic processes, and that strict assignment to specific grades can be questionable in some cases. Therefore, it is difficult to determine whether instances of misclassification are due to the lipidomic data or the histological assignment. Significant alterations were only observed in a few lipidomic features between LGG and HGG. This can be explained either by the relatively small size of the studied cohort and high diversity among the samples on the one hand, or by the dynamics of the malignancy process on the other, which renders differences in the levels of individual lipid species irrelevant (Table S5). Interestingly, the upregulation of LPCs could indicate alterations in the pathway responsible for the production of LPCs from PCs via the action of phospholipases [44]. In the next step, the LPCs are further modified and are acetylated back to PCs via lysophospholipids acetyltransferase. This cycle of hydrolysis and acetylation of phospholipids is known as the Lands cycle [44]. Other alterations in the lipid profile are related to changes in cell membrane composition and signal transduction [5,6]. Cells membranes consist of a bilayer of lipids that are distributed unevenly [45]. The outer layer is mainly composed of PCs and SMs, while the inner layer is largely comprised of PEs and phosphatidylserines (PSs). During apoptosis, PSs are transferred to the outer layer and act as labels for the cells, which undergo phagocytosis [46]. Moreover, since the membrane’s fluidity and ability to adapt to dynamic changes is associated with changes in lipid composition, changes in membrane composition may be an indicator of ongoing carcinogenesis [47]. It has previously been reported that, apart from changes in the phospholipid content, neoplasms also exhibit alterations in their sphingolipid content [42,48]. In our study, the HGG samples showed significantly decreased levels of SM 42:1;O2; however, the biochemical analysis of this observation may be vague due to the detection of only one sphingolipid showing statistically significant change (Table S5). SMs are responsible for the formation of lipid rafts in membranes and signal transduction between the cells. Furthermore, higher SM content, which leads to impaired cell-cell and cell-matrix interactions, is also often observed in cancerous tissues [49,50]. Hence, although these reports do not support the observations in this work, the lack of significant changes in other SMs and ceramides (being central molecules in SM synthesis) do not merit further discussion of this phenomena [49]. 

IDH 1/2 mutation is one of the most important factors in the classification of gliomas, as the detection of its presence is correlated with better patient prognoses [25]. Therefore, we analyzed the lipidome composition in tumors with and without this mutation, with findings revealing a distinctive set of tentative lipids in the studied model (Figure 5, Tables S4 and S6). This result may indicate that IDH 1/2 mutation not only leads to changes in the tricarboxylic acid cycle (TCA) pathway and oncometabolite accumulation, but that it may also initiate molecular alterations in the lipid profile [13,24,42,51,52] (Table S6). Similar to higher-grade gliomas, our findings showed that patients with worse outcomes (IDHw) had significantly upregulated LPCs, which can be related to alterations in the Lands cycle [44]. Interestingly, this assumption is even stronger here than in the analysis of the relationship between grade and lipidome composition because, along with the increase of LPCs, the significant downregulation of PCs in IDHw samples can be observed (Table S6). This downregulation indicates that the level of both forms of LPC and PC is interdependent. The wildtype samples were also found to contain lower levels of all PEs, which could be also explained by disruptions in the Lands cycle. However, changes in membrane composition or signal transduction are also possible explanations for this observation. The literature contains reports of alterations to the sphingolipid pathways in IDHm, which is consistent with our observations [42]. Additionally, elevated levels of SM 43:4;O2 and LacCer 42:2;O2 were also observed in the IDH wildtype samples. As mentioned earlier, SMs are responsible for cell–cell and cell–matrix interactions. Cancerous tissue is usually characterized by the upregulation of this lipid class, which causes the cell to lose control of its metabolism. Increases in SM content are usually related to the downregulation of ceramides. Even though no significant decrease in Cer was observed, the upregulation of LacCer was detected. Cancerous tissue usually contains higher concentrations of LacCer due to the higher activity of ceramide metabolic enzymes such as glucosylceramide synthase in these tissues [53]. These enzymes change Cer into LacCer, which decreases the level of Cer and, as a result, lowers the proapoptotic activity of ceramides. The higher levels of LacCer and SM could be related to the aforementioned decrease in Cer levels. However, since the direct alteration of Cer levels was not observed in this study, this relationship should be further investigated via more comprehensive instrumental analysis (e.g., reversed-phase liquid chromatography). Moreover, it should be noted that almost all LGG were IDHm, while the majority of HGG were IDHw, which could be a limitation in drawing solid conclusions. Thus, bigger study group should be employed to study this phenomenon.

Chromosomal aberrations in the 1p/19q positions can be used to differentiate oligodendrogliomas from astrocytomas, although its value is mainly diagnostic [25]. The literature also contains reports of better radiotherapy outcomes for patients with this aberration; however, evidence supporting these outcomes is not solid at present [54]. In the current study, significant alterations in lipids were observed in samples with and without 1p/19q co-deletion (Table S7). Among these lipids, the changes in phospholipids were particularly notable, as they indicate transformations in membrane bilayer composition. However, the PCA model created using tentatively selected lipids did not meet the validation criteria and could not be used to differentiate tumors based on 1p/19q co-deletion status (Figure 6). These observations could indicate that lipid metabolism is not directly related to the presence of co-deletion; however, the small study group used in this study is a limiting factor that prevents any solid conclusions. Notably, the intermediates regulating fatty acid oxidation did not indicate a relationship between this group of lipids and the presence of chromosomal aberration in previous studies profiling acylcarnitines in gliomas [24,30]. However, the literature contains studies wherein glioma samples are initially characterized based on IDH mutation, followed by the division of subsets based on the presence/absence of 1p/19q co-deletion [25,54,55,56]. This approach enables inter-tumoral heterogeneity to be narrowed down and aids in the selection of important lipids. Unfortunately, such analysis was not possible in the current study due to the small sample and the fact that 1p/19q co-deleted gliomas were all IDHm and only three non-1p/19q-deleted gliomas were IDH-mutated. Furthermore, low differentiation power could indicate the need for more complex grouping of molecular factors.

## 4. Materials and Methods

### 4.1. Chemicals and Materials

All solvents (isopropanol, methanol, water, acetonitrile) and ammonium acetate used in this research were liquid chromatography mass spectrometry (LC-MS) grade, and were purchased from Merck (Poznan, Poland). External calibrant Pierce LTQ Velos ESI Positive Ion Calibration Solution was obtained from Thermo Scientific (San Jose, CA, USA), and fibers coated with octadecyl (C18) were kindly provided by Supelco (Bellefonte, PA, USA).

### 4.2. Biological Material

Brain tumors were obtained during neurosurgical procedures in 10th Military Research Hospital and Polyclinic in Bydgoszcz. This study was approved by Bioethical Committee in Bydgoszcz (KB 628/2015).

For the intra- and inter-tumor heterogeneity experiments, eight brain tumors (four meningiomas and four gliomas) were obtained and stored at −30 °C until sampling and instrumental analysis.

The lipidome profiling experiment comprised the analysis of samples from 36 patients (22 females, 14 males) with a median age of 52 (minimum 24 and maximum 79). Of these patients, 18 had been diagnosed with meningiomas (MEN) and 18 had been diagnosed with gliomas. Sampling with the SPME probes was performed on-site directly after brain tumor excision.

A detailed characterization of the patients included in these experiments is provided in Table S8 in the Supplementary Materials.

### 4.3. Histological Data and Genetic Tests Results

The grades of the studied tumors were assessed by a histopathologist. Grade 1 and 2 tumors comprised the low-grade glioma (LGG) group, while Grade 3 and Grade 4 tumors formed the high-grade glioma (HGG) group.

The IDH1/2 status and presence of 1p/19q codeletion were assessed using a SALSA MLPA P088-D1 kit (MRC-Holland, Amsterdam, The Netherlands) in accordance with the manufacturer’s protocol.

### 4.4. Chemical Biopsy (Solid-Phase Microextraction) Protocol

Solid-phase microextraction probes were used to conduct sampling on the excised brain tumors, as shown in Figure 1. Before sampling, the fibers were trimmed to 7 mm in length and preconditioned overnight in a methanol: water (1:1 *v*/*v*) solution to activate the sorbent; the probes were then rinsed in water immediately before sampling. Extractions were performed by inserting the probe into the sample for 30 min, followed by gentle rinsing in water and storage in a freezer (−30 °C) until instrumental analysis. The fibers were desorbed into 150μl of an isopropanol:methanol (1:1 *v*/*v*) solution with the use of silanized inserts. Desorption was conducted for 1 hour under agitation at 1200 rpm. A more detailed account of this protocol, with minor modifications, has been published elsewhere [57].

### 4.5. Liquid Chromatography-High Resolution Mass Spectrometry (LC-HRMS) Analysis

Liquid chromatography coupled with high-resolution mass spectrometry (Q Exactive Focus, Thermo Scientific, Bremen, Germany) was used for instrumental analysis. The LC parameters were as follows: phase A—5 mM ammonium acetate in water; phase B—acetonitrile; gradient—0–2 min at 96% B, gradual decrease of B until 80% B at 15.0 min, and 15.1–21.0 min at 96% B; SeQuantZIC-cHILIC (Merck, Poznań, Poland) 3 µm 100 × 2.1 mm column; mobile phase flow rate—0.4 mL/min; oven temperature—40 °C; and injection volume—10 μL. The extracts obtained from the lesions sampled on-site were analyzed in full-scan mode, with lipids being tentatively identified in full MS/dd-MS2 confirmation mode. All of the samples obtained from the intra- and inter-tumor heterogeneity experiments were analyzed using full MS/dd-MS2 confirmation mode based on the list of tentative lipids.

The MS parameters were as follows: positive ion mode scan range—*m*/*z* 100–1000; acquisition via AGC (1,000,000 ions); spray voltage—1.5 kV; S-lens RF level—55%; S-lens voltage—25 V; skimmer voltage—15 V; capillary temperature—325 °C; sheath gas—60 a.u.; aux gas—30 a.u.; spare gas—2 a.u.; and probe heater temperature—320 °C. The structure of the tentatively identified lipids was confirmed based on retention time and a mass accuracy of <3ppm. Finally, the fragmentation parameters were as follows: mass resolution—35,000 FWHM; AGC target—2 × 10^4^; minimum AGC—8 × 10^3^; intensity threshold—auto; maximum IT—auto; isolation window—3.0 *m*/*z*; stepped collision energy—20 V, 30 V, 40 V; loop count—2; and dynamic exclusion—auto.

### 4.6. Data Processing

Data acquisition was performed using dedicated Thermo Scientific software, namely Xcalibur 4.2 and Free Style 1.4 (Thermo Fisher Scientific, San Jose, CA, USA). The data for the lipidomic studies was processed using LipidSearch 4.1.30 (Thermo Fisher Scientific, San Jose, CA, USA) with its accuracy set to 3 ppm and intensity threshold set to 10,000. The searched ion adducts included H^+^, NH_4_^+^, and Na^+^, with an m-score of 10 and a retention time tolerance of 0.25 being used as the alignment settings. The obtained results were then filtered using the following parameters: for extraction quality control (QC), an area coefficient of variation (CV) below 30 and not equal to 0; the QC: extraction blank area ratio above 20; and a peak quality factor above 0.85 for at least one of the studied groups. Further information relating to these search parameters has been detailed in a previous work [21]. After filtering the results, the peak areas for all of the detected tentative lipids were normalized on the summary peak area of the probe, followed by the application of statistical analysis.

### 4.7. Statistical Analysis

Statistical significance was calculated based on the Mann–Whitney U Test using Statistica 13.3 PL (StatSoft, Inc., Tulsa, OK, USA) software. All chemometric calculations were performed using PLS Toolbox (Eigenvector Research Inc., Manson, WA, USA) and MatLab 2020b software (MathWorks, Natick, MA, USA). Principal component analysis (PCA) was first applied to screen for the variability/diversity of the samples, followed by the selection of discriminant analytes based on their p-value and variable importance in projection (VIP) score. Partial least squares data analysis (PLS-DA) was also applied to assess the discriminative power of selected variables. The validation parameters consisted of calculating metrics such as root-mean-squares errors of calibration (RMSEC), cross-validation (RMSECV), and R2. Permutation using the Wilcoxon test, significance test, and Rand *t*-test was also applied. The model was considered to have passed permutation when the *p*-value was lower than 0.05.

## 5. Conclusions

SPME probes have been successfully applied on previous occasions for in vivo brain studies and the metabolomic profiling of brain tumors. The current study extends the application of this simple sampling tool, which can readily and easily be applied in clinical settings, to the analysis of brain tumor lipidomes. The resultant data shows high correlation among the different probes that were applied to sample the same tumor. This finding demonstrates the possibility of utilizing a longer coating to obtain information about the lipidome of a given lesion, which is very promising with regards to potential future in vivo applications. A wide range of phospholipids and sphingolipids were extracted from meningiomas and gliomas, which enabled the clear differentiation of tumors with various histological origins. Moreover, alterations detected in the lipidome were shown to reflect the malignancy grade of the studied glioma samples, with the most important alterations being observed at the genome level (i.e., IDH mutation). Considering the recent documentation of the use of SPME devices to simultaneously sample different brain structures for metabolomic or lipidomic analysis, it can be assumed that this strategy can be used for the concomitant sampling of neoplastic lesions and normal tissue. Eventually, it will be possible to determine target biomarkers, both polar and lipid metabolites, on-site by directly coupling SPME with mass spectrometry or other analytical instrumentation, without the need for time- and solvent-consuming chromatographic separation.

## Data Availability

Not applicable.

## Supplementary Materials

The following supporting information can be downloaded at: https://www.mdpi.com/article/10.3390/ijms23073518/s1.
