# Supplementary material for: Investigating the Potential Use of Chemical Biopsy Devices to Characterize Brain Tumor Lipidomes
Source: Int J Mol Sci. 2022 Mar 24;23(7):3518. doi: 10.3390/ijms23073518 (PMC8998862; doi:10.3390/ijms23073518)
Supplement: Supplementary file 1 [file ijms-23-03518-s001.zip › ijms-1635783-supplementary.pdf]

Investigating the potential use of chemical biopsy devices to characterize brain tumor lipidomes

Joanna Bogusiewicz[1], Bogumiła Kupcewicz[2], Paulina Zofia Goryńska[1], Karol Jaroch[1], Krzysztof Goryński[1†], Marcin Birski[3], Jacek Furtak[3], Dariusz Paczkowski[3‡], Marek Harat[3,4*], Barbara Bojko[1*]

[1] Department of Pharmacodynamics and Molecular Pharmacology, Faculty of Pharmacy, Collegium Medicum in Bydgoszcz, Nicolaus Copernicus University in Torun, 85-089 Bydgoszcz, Poland; j.bogusiewicz@cm.umk.pl (J.B.); gorynska@cm.umk.pl (P.Z.G); karol.jaroch@cm.umk.pl (K.J.); goryn-ski@cm.umk.pl (K.G.), bbojko@cm.umk.pl (B.B.)

[2] Department of Inorganic and Analytical Chemistry, Faculty of Pharmacy, Collegium Medicum in Bydgoszcz, Nicolaus Copernicus University in Torun, 85-089 Bydgoszcz, Poland; kupcewicz@cm.umk.pl (B.K.)

[3] Department of Neurosurgery, 10th Military Research Hospital and Polyclinic, 85-681 Bydgoszcz, Poland; mbir-ski@poczta.fm (M.B.); jacek.furtak2019@gmail.com (J.F.); darek_paczkowski@vp.pl (D.P.) ; ha-rat@10wsk.mil.pl (M.H.)

[4] Department of Neurosurgery and Neurology, Faculty of Health Sciences, Collegium Medicum in Bydgoszcz, Nicolaus Copernicus University in Torun, 85-168 Bydgoszcz, Poland

* Correspondence: bbojko@cm.umk.pl (B.B.) and harat@10wsk.mil.pl (M.H.)

† current affiliation: Department of Neurosurgery and Neurology, Jan Biziel University Hospital Collegium Medicum, Nicolaus Copernicus University, Bydgoszcz, Poland

‡ Current address: Bioanalysis Scientific Group, Faculty of Pharmacy, Collegium Medicum in Bydgoszcz, Nicolaus Copernicus University in Torun, 85-089 Bydgoszcz, Poland

SUPLEMENTARY MATERIALS

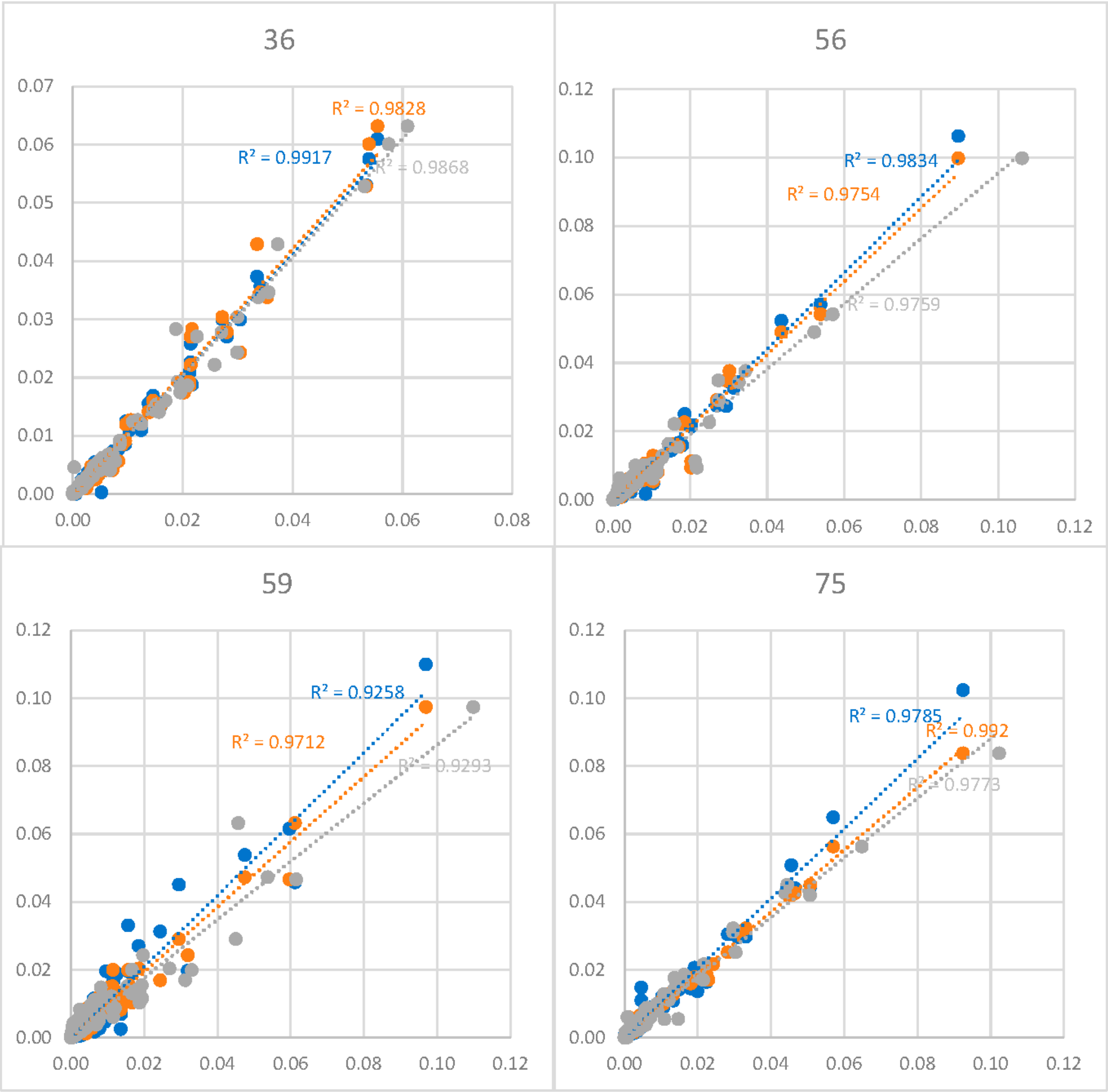

Figure S1. Plots presenting correlation factors of lipidome obtained using three different SPME probes in four different meningioma samples. Blue dots – correlation of fiber 1 and fiber 2; orange dots – correlation of fiber 1 and fiber 3; grey dots – correlation of fiber 2 and fiber 3. Axes represent normalized peak areas for subsequent lipids extracted by studied probes.

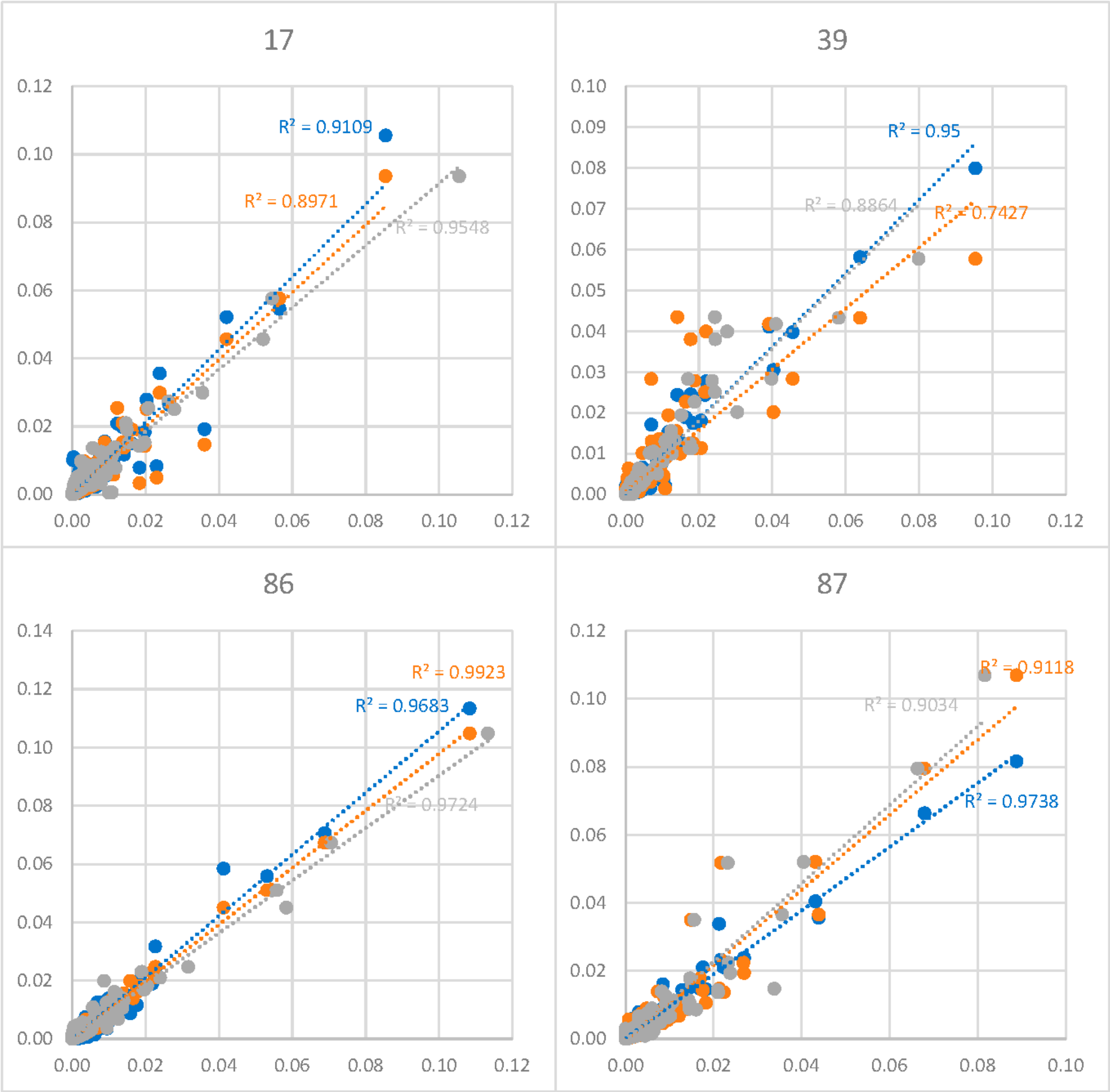

Figure S2. Plots presenting correlation factors of lipidome obtained using three different SPME probes in four glioma samples. Blue dots – correlation of fiber 1 and fiber 2; orange dots – correlation of fiber 1 and fiber 3; grey dots – correlation of fiber 2 and fiber 3. Axes represent normalized peak areas for subsequent lipids extracted by studied probes.

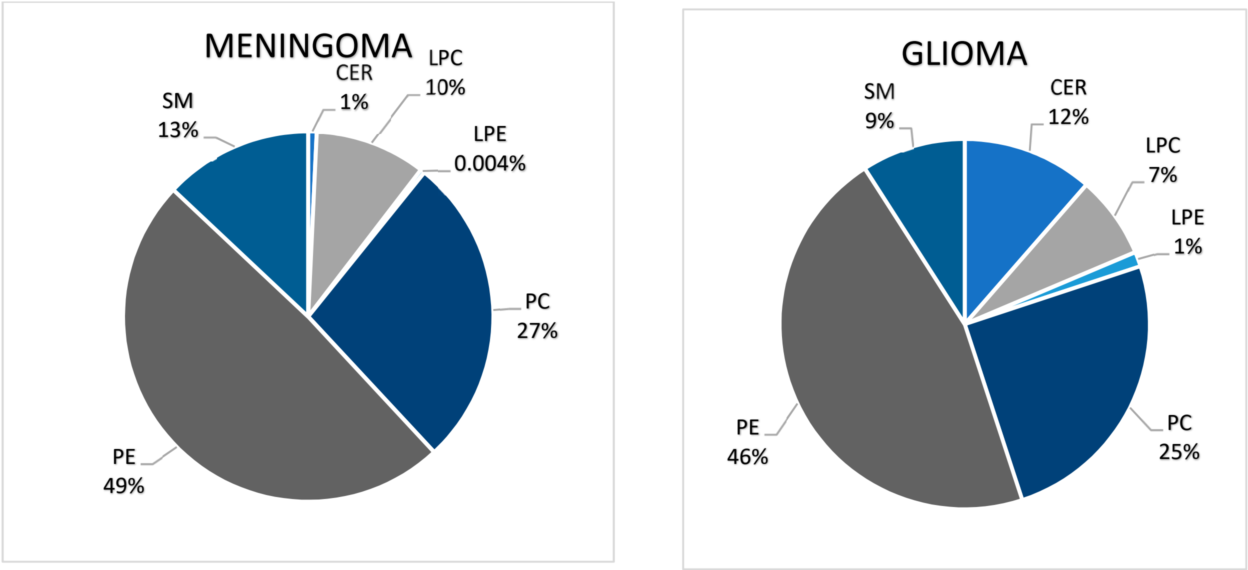

(A) **(B)**

Figure S3. Pie charts presenting lipidome profile of (A) meningiomas; (B) gliomas. *CER – ceramides and its deriviates; LPC – lysophosphatidylcholines; LPE – lysophosphatidylethanolamines; PC – phosphatidylcholines; PE – phosphatidylethanolamines; SM – sphingomyelins*

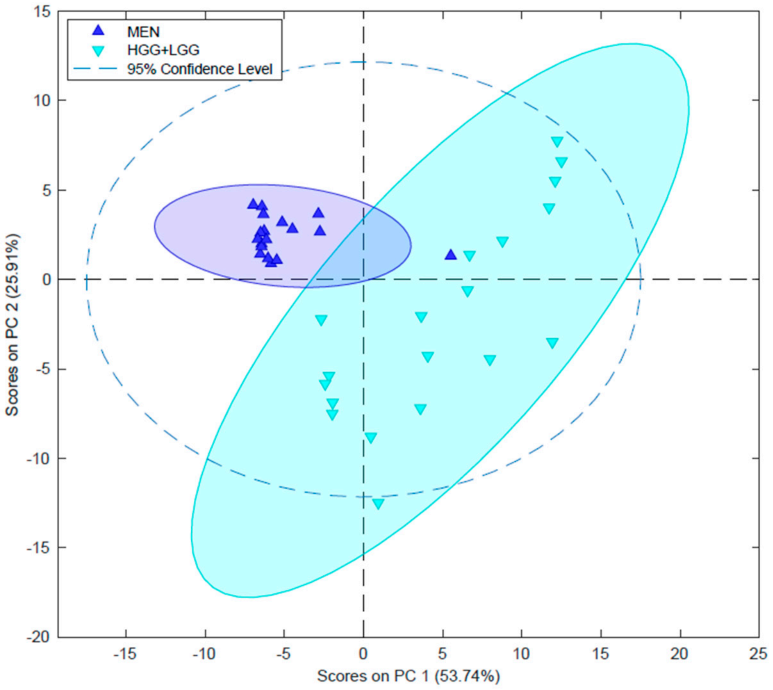

Figure S4. Principal component analysis (PCA) of brain tumors with different histological type. *HGG – high grade glioma; LGG – low grade glioma; MEN – meningioma*

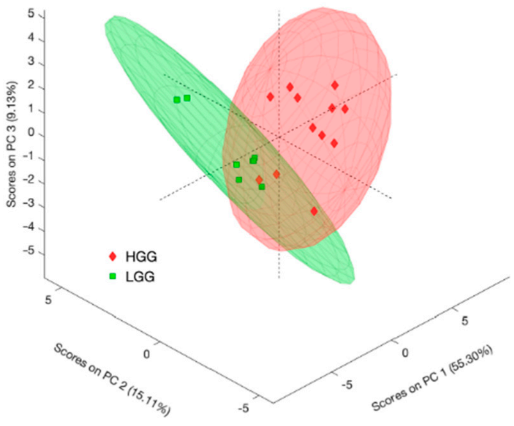

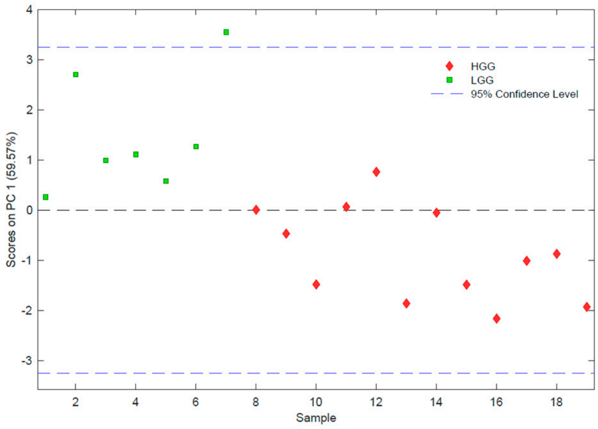

(A) **(B)**

Figure S5. Principal component analysis (PCA) of gliomas with different grade. (A) Three-dimensional principal component analysis of gliomas with different grade performed on significantly altered features. (B) One-dimensional principal component analysis of gliomas with different grade performed on significantly altered features with VIP above 1.0. *HGG – high grade glioma; LGG – low grade glioma*

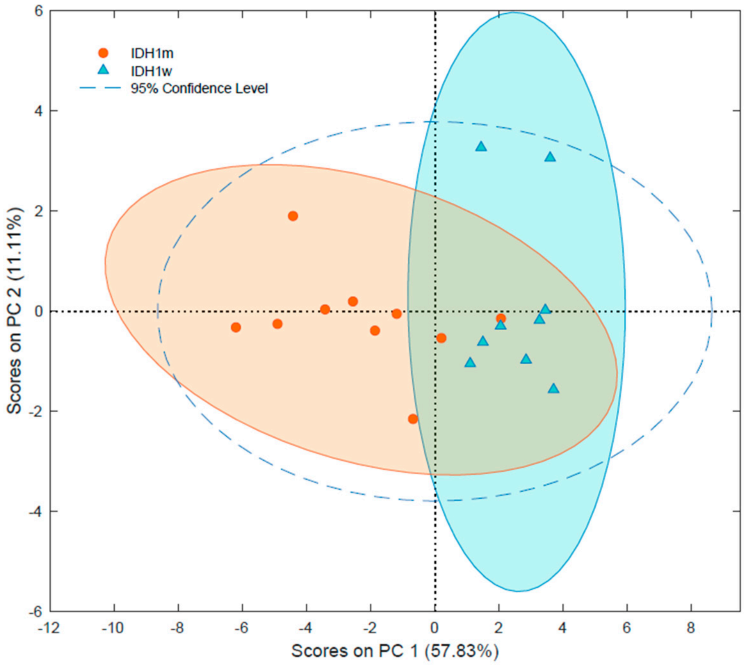

Figure S6. Principal component analysis (PCA) of gliomas with different IDH 1/2 mutation status. *IDHm – isocitrate dehydrogenase gene mutant; IDHw – isocitrate dehydrogenase gene wiltype*

Table S1. The list of lipids with confirmed structure which were detected in heterogeneity test

**Lipid Species**			**QC**		**MEN**										**HGG**									
**Shorthand**	**Adduct**	**m/z**			**36**		**56**		**59**		**75**		**MEN**		**17**		**39**		**86**		**87**		**HGG**	
			**Average***	**CV**	**Average***	**CV**	**Average***	**CV**	**Average***	**CV**	**Average***	**CV**	**Average***	**CV**	**Average***	**CV**	**Average***	**CV**	**Average***	**CV**	**Average***	**CV**	**Average***	**CV**
LPE P-16:0	+Na	460.2798	5.21E-03	9%	7.74E-03	4%	5.92E-03	9%	5.03E-03	19%	6.23E-03	6%	**6.23E-03**	**18%**	7.58E-03	74%	4.24E-03	7%	5.42E-03	19%	6.13E-03	49%	**5.84E-03**	**52%**
Cer 42:3; O2	+H	646.6133	2.46E-04	16%	3.45E-04	11%	4.19E-04	16%	2.80E-04	14%	2.76E-04	15%	**3.30E-04**	**22%**	4.43E-04	23%	2.89E-04	33%	3.91E-04	26%	1.89E-04	11%	**3.28E-04**	**39%**
HexCer 36:2; O2	+H	726.5878	3.40E-04	13%	-	-	7.08E-07	173%	2.62E-06	87%	2.26E-06	173%	**1.40E-06**	**164%**	1.77E-03	33%	5.14E-04	40%	9.61E-04	68%	1.13E-05	31%	**8.15E-04**	**95%**
HexCer 38:1; O2	+H	756.6348	2.25E-04	12%	3.32E-06	98%	6.46E-06	33%	4.44E-06	14%	-	-	**3.56E-06**	**84%**	1.60E-03	31%	1.73E-04	73%	3.36E-04	79%	2.11E-05	173%	**5.34E-04**	**131%**
HexCer 41:1; O2	+H	798.6817	1.02E-03	6%	4.76E-05	16%	1.05E-05	119%	6.87E-05	77%	3.66E-05	56%	**4.08E-05**	**81%**	5.73E-03	26%	4.73E-06	88%	1.45E-03	76%	9.40E-06	106%	**1.80E-03**	**143%**
HexCer 42:2; O2	+H	810.6817	4.03E-04	10%	4.55E-04	164%	6.38E-04	170%	8.93E-05	88%	3.35E-04	163%	**3.80E-04**	**170%**	2.37E-03	24%	3.43E-04	164%	7.88E-04	78%	1.69E-04	145%	**9.18E-04**	**110%**
HexCer 43:2; O2	+H	824.6974	6.28E-05	15%	-	-	1.37E-06	173%	2.80E-07	173%	5.71E-06	173%	**1.84E-06**	**270%**	4.86E-04	49%	-	-	1.54E-04	89%	5.48E-06	173%	**1.61E-04**	**147%**
HexCer 44:2; O2	+H	838.7130	2.31E-03	4%	-	-	1.68E-06	93%	4.64E-04	68%	7.39E-05	171%	**1.35E-04**	**184%**	1.15E-02	27%	-	-	2.58E-03	76%	2.89E-06	173%	**3.52E-03**	**147%**
LPC P-16:0	+H	480.3449	2.73E-03	12%	2.24E-03	8%	6.01E-03	13%	2.23E-03	14%	2.73E-03	4%	**3.30E-03**	**51%**	5.50E-03	31%	1.34E-03	20%	1.84E-03	38%	1.31E-03	36%	**2.50E-03**	**80%**
LPC O-16:0	+H	482.3605	1.30E-03	15%	4.65E-04	13%	1.19E-03	13%	6.59E-04	37%	4.13E-04	5%	**6.81E-04**	**50%**	3.75E-03	40%	6.89E-04	27%	2.38E-03	9%	1.53E-03	40%	**2.09E-03**	**66%**
LPC 16:1	+H	494.3241	3.42E-03	8%	2.99E-04	19%	1.69E-03	37%	3.95E-04	12%	8.58E-04	88%	**8.11E-04**	**88%**	2.73E-03	134%	1.21E-02	66%	8.70E-03	29%	9.82E-03	14%	**8.34E-03**	**64%**
LPC 16:0	+H	496.3398	2.59E-02	11%	1.38E-02	8%	1.84E-02	4%	1.77E-02	13%	1.62E-02	8%	**1.65E-02**	**13%**	3.76E-02	34%	4.89E-02	21%	4.19E-02	15%	5.97E-02	17%	**4.70E-02**	**26%**
LPC P-18:0	+H	508.3762	1.52E-03	12%	5.52E-04	16%	9.09E-04	11%	6.83E-04	16%	6.85E-04	12%	**7.07E-04**	**22%**	2.45E-03	46%	7.26E-04	31%	3.42E-03	90%	1.72E-03	26%	**2.08E-03**	**84%**
LPC 18:3	+H	518.3241	1.51E-02	5%	8.07E-03	7%	1.10E-02	5%	9.79E-03	9%	9.64E-03	12%	**9.61E-03**	**13%**	2.20E-02	41%	3.15E-02	19%	2.52E-02	8%	3.71E-02	22%	**2.90E-02**	**29%**
LPC 18:2	+H	520.3398	2.84E-03	13%	4.43E-04	12%	3.16E-03	7%	1.74E-03	36%	2.92E-04	56%	**1.41E-03**	**88%**	1.04E-03	53%	1.97E-03	7%	9.51E-03	19%	6.03E-03	36%	**4.64E-03**	**81%**
LPC 18:1	+H	522.3554	1.46E-02	8%	6.05E-03	10%	1.45E-02	13%	6.21E-03	7%	9.26E-03	22%	**9.00E-03**	**42%**	2.00E-02	28%	2.14E-02	35%	3.36E-02	18%	2.12E-02	16%	**2.40E-02**	**32%**
LPC 18:0	+H	524.3711	8.85E-03	5%	6.53E-03	6%	1.12E-02	12%	8.38E-03	22%	6.98E-03	5%	**8.27E-03**	**26%**	1.98E-02	75%	7.47E-03	18%	8.64E-03	21%	1.28E-02	41%	**1.22E-02**	**69%**
PC 30:0	+Na	728.5201	1.13E-02	2%	9.23E-03	6%	1.36E-02	11%	9.68E-03	17%	1.00E-02	3%	**1.06E-02**	**19%**	1.19E-02	19%	1.83E-02	5%	1.37E-02	22%	6.87E-03	31%	**1.27E-02**	**37%**
PC P-34:1	+H	744.5902	1.94E-02	8%	2.06E-02	6%	3.21E-02	7%	1.76E-02	38%	2.60E-02	10%	**2.41E-02**	**27%**	3.70E-02	21%	3.22E-03	7%	1.38E-02	47%	1.59E-03	47%	**1.39E-02**	**111%**
PC 32:1	+Na	754.5357	2.21E-02	8%	5.51E-03	16%	1.74E-02	6%	1.16E-02	13%	1.38E-02	6%	**1.21E-02**	**38%**	3.29E-02	22%	5.51E-02	24%	3.21E-02	4%	3.39E-02	11%	**3.85E-02**	**31%**
PC 32:0	+Na	756.5514	7.25E-02	2%	9.70E-02	3%	5.15E-02	10%	8.90E-02	15%	5.44E-02	6%	**7.30E-02**	**30%**	5.18E-02	6%	7.95E-02	13%	8.91E-02	12%	7.57E-02	6%	**7.40E-02**	**21%**
PC 34:3	+H	756.5538	7.08E-02	8%	9.08E-02	6%	4.81E-02	5%	8.17E-02	26%	5.47E-02	6%	**6.89E-02**	**30%**	4.63E-02	11%	7.43E-02	9%	8.13E-02	22%	7.51E-02	7%	**6.93E-02**	**24%**
PC P-36:4	+H	766.5745	1.70E-02	5%	1.80E-02	4%	3.17E-02	14%	1.60E-02	28%	2.04E-02	9%	**2.15E-02**	**32%**	2.35E-02	9%	7.38E-03	19%	8.96E-03	42%	3.82E-03	21%	**1.09E-02**	**74%**
PC 34:2	+Na	780.5514	2.37E-02	2%	6.89E-03	22%	1.60E-02	9%	2.50E-02	23%	9.68E-03	27%	**1.44E-02**	**54%**	2.01E-02	30%	2.53E-02	18%	4.07E-02	4%	3.03E-02	25%	**2.91E-02**	**32%**
PC 34:1	+Na	782.5670	2.08E-01	2%	1.21E-01	5%	1.84E-01	2%	2.00E-01	2%	2.14E-01	8%	**1.80E-01**	**21%**	2.19E-01	2%	1.84E-01	14%	2.33E-01	3%	2.25E-01	13%	**2.15E-01**	**12%**
PC 36:4	+Na	804.5514	1.31E-02	6%	5.66E-03	15%	9.64E-03	8%	1.35E-02	22%	1.29E-02	19%	**1.04E-02**	**35%**	1.16E-02	15%	1.71E-02	18%	1.17E-02	20%	2.00E-02	3%	**1.51E-02**	**28%**
PC 36:3	+Na	806.5670	1.35E-02	3%	3.48E-03	13%	1.09E-02	15%	1.58E-02	9%	9.03E-03	13%	**9.80E-03**	**48%**	1.25E-02	14%	1.47E-02	7%	1.80E-02	10%	2.10E-02	7%	**1.66E-02**	**22%**
PC 36:2	+Na	808.5827	2.55E-02	3%	7.90E-03	18%	2.27E-02	4%	2.34E-02	19%	1.83E-02	16%	**1.81E-02**	**38%**	2.58E-02	10%	2.30E-02	16%	3.92E-02	6%	2.54E-02	16%	**2.84E-02**	**25%**
PC 36:1	+Na	810.5983	5.32E-02	8%	2.98E-02	4%	7.35E-02	13%	4.84E-02	25%	6.64E-02	10%	**5.45E-02**	**35%**	5.81E-02	9%	3.32E-02	21%	3.94E-02	21%	4.11E-02	11%	**4.29E-02**	**26%**
PC 38:5	+Na	830.5670	3.68E-03	3%	1.27E-03	9%	2.66E-03	18%	4.15E-03	20%	3.59E-03	20%	**2.92E-03**	**43%**	3.67E-03	13%	5.51E-03	22%	4.18E-03	17%	5.11E-03	17%	**4.62E-03**	**23%**
PC 38:4	+Na	832.5827	1.12E-02	7%	6.39E-03	13%	1.45E-02	14%	1.47E-02	36%	1.60E-02	28%	**1.29E-02**	**39%**	1.03E-02	8%	9.41E-03	4%	8.16E-03	13%	1.06E-02	16%	**9.63E-03**	**14%**
PE P-34:2	+H	700.5276	1.37E-03	8%	1.85E-03	7%	1.90E-03	10%	1.93E-03	23%	1.14E-03	8%	**1.70E-03**	**24%**	1.43E-03	26%	6.91E-04	62%	1.37E-03	17%	3.90E-04	59%	**9.71E-04**	**56%**
PE P-34:1	+H	702.5432	3.61E-02	8%	7.02E-02	7%	3.98E-02	7%	3.64E-02	17%	6.13E-02	6%	**5.19E-02**	**30%**	3.49E-02	17%	1.01E-02	7%	1.46E-02	37%	6.47E-03	23%	**1.65E-02**	**73%**
PE 34:2	+H	716.5225	1.50E-03	13%	1.41E-03	20%	2.24E-03	11%	2.68E-03	34%	7.37E-04	6%	**1.77E-03**	**50%**	4.84E-04	25%	9.33E-04	43%	1.64E-03	15%	1.59E-03	7%	**1.16E-03**	**47%**
PE 34:1	+H	718.5381	1.23E-02	7%	2.18E-02	6%	1.93E-02	6%	1.53E-02	18%	1.78E-02	6%	**1.85E-02**	**16%**	3.11E-03	30%	8.78E-03	4%	7.99E-03	14%	1.25E-02	48%	**8.10E-03**	**54%**
PE P-34:1	+Na	724.5252	4.36E-02	17%	7.92E-02	24%	4.24E-02	6%	3.69E-02	17%	6.92E-02	17%	**5.69E-02**	**37%**	4.06E-02	18%	4.39E-02	8%	1.47E-02	35%	2.16E-02	18%	**3.02E-02**	**45%**

PE 34:2	+Na	738.5044	1.59E-03	19%	1.54E-03	10%	2.17E-03	21%	2.77E-03	30%	8.02E-04	7%	**1.82E-03**	**48%**	6.59E-04	35%	1.42E-03	14%	1.74E-03	12%	1.80E-03	9%	**1.41E-03**	**36%**
PE 34:1	+Na	740.5201	1.62E-02	11%	2.66E-02	10%	2.37E-02	4%	1.76E-02	19%	1.89E-02	4%	**2.17E-02**	**19%**	4.19E-03	44%	1.32E-02	13%	1.01E-02	19%	1.73E-02	27%	**1.12E-02**	**49%**
PE 36:2	+H	744.5538	5.19E-03	17%	3.05E-03	11%	8.68E-03	9%	6.02E-03	14%	2.69E-03	4%	**5.11E-03**	**51%**	2.28E-03	38%	3.13E-03	19%	6.96E-03	17%	4.74E-03	37%	**4.28E-03**	**49%**
PE P-38:7	+H	746.5119	2.18E-02	11%	4.10E-02	5%	2.64E-02	20%	2.32E-02	33%	2.22E-02	23%	**2.82E-02**	**32%**	6.11E-03	48%	3.68E-02	23%	4.66E-03	39%	2.20E-02	15%	**1.74E-02**	**82%**
PE 36:1	+H	746.5694	7.71E-03	48%	1.22E-02	78%	3.09E-02	6%	8.81E-03	48%	1.39E-02	70%	**1.65E-02**	**66%**	1.81E-02	45%	1.66E-02	90%	7.87E-03	12%	7.74E-03	6%	**1.26E-02**	**70%**
PE O-36:4	+Na	748.5252	8.38E-03	11%	1.24E-02	19%	5.21E-03	8%	9.01E-03	27%	1.24E-02	19%	**9.75E-03**	**37%**	6.06E-03	47%	1.37E-02	26%	6.29E-03	6%	1.24E-02	13%	**9.62E-03**	**43%**
PE P-38:6	+H	748.5276	8.38E-03	11%	1.24E-02	19%	5.21E-03	8%	8.85E-03	26%	1.24E-02	19%	**9.70E-03**	**37%**	6.06E-03	47%	1.37E-02	26%	6.29E-03	6%	1.39E-02	2%	**1.00E-02**	**45%**
PE P-38:5	+H	750.5432	2.21E-02	4%	2.55E-02	12%	1.52E-02	11%	1.73E-02	16%	2.44E-02	7%	**2.06E-02**	**25%**	3.81E-02	38%	1.41E-02	10%	1.75E-02	78%	9.92E-03	2%	**1.99E-02**	**71%**
PE 36:2	+Na	766.5357	7.97E-03	3%	4.94E-03	13%	1.24E-02	4%	9.11E-03	5%	4.78E-03	11%	**7.80E-03**	**43%**	3.84E-03	41%	8.20E-03	4%	1.06E-02	13%	1.08E-02	35%	**8.36E-03**	**41%**
PE 38:5	+H	766.5381	8.02E-03	3%	4.95E-03	12%	1.24E-02	4%	9.10E-03	6%	4.79E-03	11%	**7.80E-03**	**43%**	3.85E-03	41%	8.20E-03	10%	1.06E-02	13%	1.05E-02	37%	**8.30E-03**	**42%**
PE 36:1	+Na	768.5514	3.96E-02	24%	4.44E-02	6%	5.91E-02	28%	3.54E-02	7%	2.52E-02	49%	**4.10E-02**	**38%**	1.78E-02	39%	3.14E-02	8%	1.99E-02	30%	3.21E-02	24%	**2.53E-02**	**34%**
PE P-40:7	+H	774.5432	2.21E-02	9%	4.21E-02	4%	1.42E-02	16%	2.35E-02	31%	2.44E-02	18%	**2.61E-02**	**43%**	2.06E-02	34%	3.35E-02	36%	1.47E-02	27%	1.96E-02	12%	**2.21E-02**	**44%**
PE P-38:2	+Na	778.5721	5.70E-03	8%	5.51E-03	2%	1.39E-03	11%	5.28E-03	14%	4.75E-03	18%	**4.23E-03**	**43%**	1.31E-02	24%	5.03E-03	20%	6.65E-03	38%	3.80E-03	15%	**7.15E-03**	**58%**
PE P-40:5	+H	778.5745	5.80E-03	5%	5.65E-03	2%	1.36E-03	7%	5.34E-03	17%	4.76E-03	17%	**4.28E-03**	**44%**	1.32E-02	24%	5.32E-03	25%	6.74E-03	40%	3.83E-03	16%	**7.28E-03**	**57%**
PE 38:4	+Na	790.5357	1.97E-02	8%	2.66E-02	10%	3.04E-02	6%	2.07E-02	18%	1.56E-02	6%	**2.33E-02**	**27%**	5.50E-03	52%	1.61E-02	16%	8.39E-03	16%	1.80E-02	32%	**1.20E-02**	**52%**
PE 38:1	+Na	796.5827	9.15E-03	6%	4.82E-03	8%	8.58E-03	12%	9.57E-03	12%	8.31E-03	5%	**7.82E-03**	**25%**	1.31E-02	9%	7.22E-03	21%	8.37E-03	8%	8.05E-03	15%	**9.18E-03**	**28%**
PE P-40:6	+Na	798.5408	1.56E-02	25%	1.46E-02	15%	9.55E-03	18%	2.13E-02	12%	2.09E-02	8%	**1.66E-02**	**32%**	1.59E-02	25%	1.48E-02	13%	1.88E-02	11%	1.75E-02	22%	**1.68E-02**	**18%**
PE P-40:4	+Na	802.5721	4.27E-03	8%	6.00E-03	1%	9.03E-04	11%	4.79E-03	35%	3.18E-03	24%	**3.72E-03**	**58%**	7.19E-03	23%	4.87E-03	33%	4.08E-03	48%	2.66E-03	18%	**4.70E-03**	**46%**
PE 40:6	+Na	814.5357	2.68E-03	4%	7.62E-04	9%	1.13E-04	49%	7.41E-03	51%	1.89E-03	13%	**2.54E-03**	**134%**	8.47E-04	53%	7.32E-04	48%	1.48E-03	10%	2.14E-03	78%	**1.30E-03**	**74%**
SM 42:4; O2	+H	809.6531	1.78E-02	5%	4.99E-02	6%	1.96E-02	21%	3.11E-02	44%	3.53E-02	11%	**3.40E-02**	**38%**	4.89E-03	12%	5.05E-03	51%	4.94E-03	27%	4.36E-03	46%	**4.81E-03**	**32%**
SM 43:4; O2	+H	823.6688	4.26E-03	6%	9.66E-03	3%	3.55E-03	19%	5.96E-03	42%	5.22E-03	14%	**6.10E-03**	**43%**	6.63E-03	2%	1.04E-03	56%	2.19E-03	35%	1.17E-03	22%	**2.76E-03**	**88%**
ST 42:2; O2	+H	890.6386	1.69E-04	19%	-	-	-	-	3.43E-06	101%	8.68E-07	173%	**1.08E-06**	**203%**	7.52E-04	14%	2.68E-06	173%	2.73E-04	77%	-	-	**2.57E-04**	**131%**

***Average peak area after on-fiber normalization**

CV – coefficient of variation; HGG – high grade glioma; m/z – mass to charge ratio; MEN -meningioma; QC – quality control; RT – retention time

Table S2. The list of lipid species identified with the use of MS/MS mode

m/z	RT	adduct	Lipid Species Shorthand
620.5976	4.98	+H	Cer 40:2;O2
666.6395	4.96	+H	Cer 42:1;O3
646.6133	4.64	+H	Cer 42:3;O2
662.6446	2.93	+H	Cer 43:2;O2
674.6446	4.83	+H	Cer 44:3;O2
728.6035	3.01	+H	HexCer 36:1;O2
726.5878	5.31	+H	HexCer 36:2;O2
756.6348	3.2	+H	HexCer 38:1;O2
800.6610	4.92	+H	HexCer 40:1;O3
782.6504	4.93	+H	HexCer 40:2;O2
798.6817	2.89	+H	HexCer 41:1;O2
814.6767	4.99	+H	HexCer 41:1;O3
796.6661	4.85	+H	HexCer 41:2;O2
794.6504	5.16	+H	HexCer 41:3;O2
812.6974	2.77	+H	HexCer 42:1;O2
828.6923	4.95	+H	HexCer 42:1;O3
810.6817	3.02	+H	HexCer 42:2;O2
810.6817	4.93	+H	HexCer 42:2;O2
826.6767	4.68	+H	HexCer 42:2;O3
808.6661	4.74	+H	HexCer 42:3;O2
842.7080	4.87	+H	HexCer 43:1;O3
824.6974	2.62	+H	HexCer 43:2;O2
838.7130	2.57	+H	HexCer 44:2;O2
836.6974	4.92	+H	HexCer 44:3;O2
862.6250	8.25	+H	LacCer 34:1;O2
972.7346	7.92	+H	LacCer 42:2;O2
496.3398	8.57	+H	LPC 16:0
482.3605	8.65	+H	LPC O-16:0
480.3449	8.13	+H	LPC P-16:0
494.3241	8.27	+H	LPC 16:1
524.3711	8.24	+H	LPC 18:0
508.3762	8.56	+H	LPC P-18:0
522.3554	8.38	+H	LPC 18:1
520.3398	8.16	+H	LPC 18:2
518.3217	8.65	+H	LPC 18:3
544.3374	8.41	+H	LPC 20:4
460.2798	9.7	+Na	LPE P-16:0
634.4806	8.16	+H	PC 26:1
728.5201	6.39	+Na	PC 30:0
756.5514	6.39	+Na	PC 32:0
754.5357	6.11	+Na	PC 32:1
782.5670	6.65	+Na	PC 34:1
744.5902	5.9	+H	PC P-34:1
780.5514	6.24	+Na	PC 34:2
756.5538	6.76	+H	PC 34:3
738.5432	6.96	+H	PC P-34:4
810.5983	6.46	+Na	PC 36:1
808.5827	6.59	+Na	PC 36:2
806.5670	6.27	+Na	PC 36:3

804.5514	5.88	+Na	PC 36:4
832.5827	6.27	+Na	PC 38:4
830.5670	5.94	+Na	PC 38:5
742.5357	7.25	+Na	PE 34:0
718.5381	7.10	+H	PE 34:1
740.5201		+Na	
702.5432	7.25	+H	PE P-34:1
724.5252		+Na	
716.5225	7.35	+H	PE 34:2
738.5044		+Na	
700.5276	7.14	+H	PE P-34:2
716.5589	7.12	+H	PE P-35:1
746.5694	7.20	+H	PE 36:1
768.5514		+Na	
744.5538	7.16	+H	PE 36:2
764.5201	7.08	+Na	PE 36:3
748.5252	6.79	+Na	PE O-36:4
724.5276	7.05	+H	PE P-36:4
746.5095	6.78	+Na	PE O-36:5
766.5721	6.99	+Na	PE P-37:1
796.5827	7.05	+Na	PE 38:1
756.5902	6.88	+H	PE P-38:2
778.5721	7.41	+Na	PE O-38:3
768.5538	6.90	+H	PE 38:4
790.5357		+Na	
752.5589	6.98	+H	PE P-38:4
752.5565	6.89	+H	PE P-38:4
766.5357	6.93	+H	PE 38:5
766.5381	7.01	+H	PE 38:5
788.5201		+Na	
774.5408	7.00	+Na	PE O-38:5
750.5432	7.01	+H	PE P-38:5
786.5044	7.12	+Na	PE 38:6
772.5252	7.02	+Na	PE O-38:6
772.5252	6.83	+Na	PE O-38:6
748.5276	6.87	+H	PE P-38:6
770.5097		+Na	
746.5119	6.80	+H	PE P-38:7
784.6215	6.87	+H	PE P-40:2
804.5878	6.84	+Na	PE O-40:4
780.5902	6.95	+H	PE P-40:4
802.5721		+Na	
816.5514	6,89	+Na	PE 40:5
778.5745	6.67	+H	PE P-40:5
792.5538	6.95	+H	PE 40:6
800.5565	6.74	+Na	PE O-40:6
798.5408	6.85	+Na	PE P-40:6
774.5432	6.69	+H	PE P-40:7
806.6058	6.87	+H	PE P-42:5
809.6531	7.42	+H	SM 32:4;O2
835.6688	7.27	+H	SM 34:5;O2

725.5592	7.59	+H	SM 36:4;O2
753.5905	7.41	+H	SM 38:4;O2
781.6218	7.52	+H	SM 40:4;O2
801.6844	7.21	+H	SM 41:1;O2
799.6688	7.43	+H	SM 41:2;O2
815.7001	7.11	+H	SM 42:1;O2
811.6688	7.18	+H	SM 42:3;O2
829.7157	7.11	+H	SM 43:1;O2
827.7001	7.91	+H	SM 43:2;O2
823.6663	7.4	+H	SM 43:4;O2
841.7157	7.16	+H	SM 44:2;O2
890.6386	2.37	+H	ST 42:2;O2

m/z – mass to charge ratio; RT – retention time

Table S3. The lipidome composition of meningiomas and gliomas based on MS/MS confirmed groups of lipids.

Lipid group	Meningioma		Glioma		ratio [glioma: meningioma]	U Mann Whitney test
	Average*	CV	Average*	CV		
Cer	2,30E-05	332%	1,16E-03	167%	50,42	**0,02**
HexCer	1,98E-03	274%	3,22E-02	150%	16,25	**0,02**
LacCer	4,06E-04	147%	3,47E-04	91%	0,85	0,77
LPC	3,16E-02	114%	2,13E-02	43%	0,67	0,23
LPE P-16:0	1,22E-03	37%	3,80E-03	76%	3,10	**<0.01**
PC	8,92E-02	10%	7,39E-02	30%	0,83	**0,02**
PE	1,60E-01	10%	1,36E-01	17%	0,85	**<0.01**
SM	4,24E-02	16%	2,68E-02	36%	0,63	**<0.01**
ST 42:2; O2	2,61E-04	31%	8,71E-05	98%	0,33	**<0.01**

***Average peak area after on-fiber normalization**

CV – coefficient of variation; Cer – ceramides; HexCer – hexosyl ceramides; HGG – high grade glioma; LacCer – lactosyl ceramides; LGG – low grade glioma; LPC – lysophosphatidylcholines; LPE – lysophosphatidylethanolamines; MEN – meningioma; PC – phosphatidylcholines; PE – phosphatidylethanolamines; QC – quality control; SM – sphingomyelins; ST – sulfatides

Table S4. Parameters for Partial least squares-discriminant analysis (PLS-DA).

Paramter	MEN vs glioma		Glioma grade		IDH mutation status	
Classes	MEN	LGG+HGG	LGG	HGG	IDHm	IDHw
Variable inclusion paramters	VIP-score>1		p-value<0.05<br>VIP-score>1		p-value<0.05<br>VIP-score>1	
Data preprocessing	Log10, Autoscale		Log10, Autoscale		Log10, Autoscale	
N	18	19	7	12	10	9
Number of LV	5		2		2	
RMSEC	0.084	0.084	0.260	0.260	0.184	0.184
RMSECV	0.225	0.225	0.322	0.322	0.263	0.263
R2(calibration)	0.972	0.972	0.710	0.710	0.864	0.864
R2(CV)	0.811	0.811	0.570	0.570	0.736	0.736
Permutation test	wilcoxon test – <0.05<br>significance test – <0.05<br>rand t-test – <0.05		wilcoxon test – <0.05<br>significance test – 0.056<br>rand t-test – <0.05		wilcoxon test – <0.05<br>significance test – <0.05<br>rand t-test – <0.05	

CV – coefficient of variation; HGG – high grade glioma; IDHm – isocitrate dehydrogenase gene mutant; IDHw – isocitrate dehydrogenase gene wiltype; LGG – low grade glioma; LV – latent variables; MEN – meningioma; N – number of samples; RMSEC– root mean square error of calibration; RMSECV – root mean square error of cross-validation; VIP - Variable Importance in Projection

Table S5. The list od significantly altered tentative lipids in differentiation of low and high grade gliomas (p<0.05). In italic were given tentative lipid groups for detected m/z.

m/z	Lipid Species Shorthand	HGG/LGG ratio	VIP>1
480.34485	LPC P-16:0	2.66	+
502.32680	*CerP, LPC*	3.99	-
508.37615	LPC P-18:0	2.38	-
520.33977	LPC 18:2	2.64	-
718.57448	*Cer, CerP, LPC, LPE, PC, PE*	2.74	-
720.55378	*CerP, PC, PE*	1.55	+
745.55727	*HexCer*	0.66	-
748.58269	*PC*	1.68	-
773.53031	*PG*	0.75	-
781.61940	*SM, CerP*	0.37	+
794.60341	*CerP, PC, PE*	2.90	-
794.60582	*PC*	2.92	-
796.61906	*CerP*	4.53	-
796.62147	*CerP*	4.72	-
800.55646	PE O-40:6	0.66	-
815.70005	SM 42:1; O2	0.37	+
818.60341	*CerP*	10.38	-

Cer – ceramides; CerP – ceramide phosphate; HexCer – hexosyl ceramides; HGG – high grade glioma; LGG – low grade glioma; LPC – lysophosphatidylcholines; LPE – lysophosphatidylethanolamines; m/z – mass to charge ratio; PC – phosphatidylcholines; PE – phosphatidylethanolamines; PG – phosphatidylglycerols; SM – sphingomyelins; VIP - Variable Importance in Projection

Table S6. The list od significantly altered tentative lipids in differentiation of IDH mutant and wildtype gliomas (p<0.05). In italic were given tentative lipid groups for detected m/z.

m/z	Lipid Species Shorthand	IDHw/ IDHm ratio	VIP>1
438.2979	*LPE*	1.98	-
460.2798	LPE P-16:0	0.56	-
482.36050	LPC O-16:0	6.15	+
502.32680	*CerP, LPC*	2.96	+
508.37615	LPC P-18:0	4.91	-
524.37107	LPC 18:0	1.89	-
628.60271	*Cholesterol ester*	1.79	+
700.5275	PE P-34:2	0.31	-
702.5432	PE P-34:1	0.54	-
706.5381	*CerP, PC, PE*	0.62	-
716.5588	PE P-35:1	0.27	-
717.59050	*CerP, SM*	1.80	+
718.5744	*Cer, CerP, LPC, LPE, PC, PE*	2.74	-
720.59017	*Cer, CerP, LPC, PE,*	2.46	+
723.5411	*SM*	1.93	-
724.5251	PE P-34:1	0.39	-
724.5275	PE P-36:4	0.43	-
725.53106	*HexCer*	1.96	+
728.52008	PC 30:0	0.47	-

728.5224	*CerP, PC, PE*	0.48	-
729.5252	*PG*	0.46	-
729.59050	*CerP, SM*	2.78	+
734.5670	*PC, PE*	0.62	-
740.5200	PE 34:1	2.21	-
742.5721	*PC*	3.11	-
742.5745	*PC*	3.05	-
744.5513	*PC*	0.33	-
745.55727	*HexCer*	0.39	+
746.5694	PE 36:1	0.25	-
746.6057	*Cer, CerP, LPC, LPE*	2.49	-
747.6093	*HexCer*	2.49	-
748.52516	PE O-36:4	0.36	+
750.5432	PE O-38:5	0.47	-
751.57245	*CerP, SM*	2.55	+
754.53573	PC 32:1	0.59	-
755.5408	*PG*	0.52	-
756.55138	PC 32:0	0.51	-
765.5252	*PG*	0.59	-
766.5745	*CerP, PC*	3.10	-
768.55378	PE 38:4	0.51	+
768.5877	*CerP, PC, PE*	4.08	-
768.59017	*PC*	4.01	+
774.5432	PE P-40:7	0.53	-
778.5721	PE O-38:3	3.38	-
785.65310	*CerP, SM*	2.03	+
786.5044	PE 38:6	0.35	-
787.6687	*CerP, SM*	1.81	-
790.5721	*PC*	7.39	-
792.5877	*PC, PE*	3.97	-
792.5901	*PC*	3.98	-
794.5670	*PC, PE*	0.48	-
794.5694	*PC, PE*	0.47	-
794.6034	*CerP, PC, PE*	3.17	-
794.6058	*PC*	3.17	-
796.58508	*PC,PE*	0.53	+
796.6190	*CerP*	5.74	-
796.6214	*CerP*	5.97	-
800.55646	PE O-40:6	0.78	+
810.59833	PC 36:1	0.60	-
812.6163	*PC*	1.53	-
813.6191	*PG*	1.58	-
814.5344	*ST*	0.35	-
814.5357	*PC*	0.38	-
814.5381	*PE*	0.37	-
818.6034	*CerP*	8,19	-
823.66635	SM 43:4; O2	1.92	+
972.73457	LacCer 42:2; O2	7.52	-

Cer – ceramides; CerP – ceramide phosphate; HexCer – hexosyl ceramides; LacCer – lactosyl ceramides; IDHm – isocitrate dehydrogenase gene mutant; IDHw – isocitrate dehydrogenase gene wiltype; LPC – lysophosphatidylcholines; LPE – lysophosphatidylethanolamines; m/z – mass to charge ratio; PC –

phosphatidylcholines; PE – phosphatidylethanolamines; PG – phosphatidylglycerols; SM – sphingomyelins; ST – sulfatides; VIP - Variable Importance in Projection

Table S7. The list od significantly altered tentative lipids in differentiation of tumors with 1p/19q co-deletion and withut this abberation (p<0.05). In italic were given tentative lipid groups for detected m/z.

m/z	Lipid Species Shorthand	n-del/del ratio
438.2979	*LPE*	2.30
480.3448	LPC P-16:0	4.62
482.3605	LPC O-16:0	5.15
508.3761	LPC P-18:0	4.79
524.3710	LPC 18:0	2.08
700.5275	PE P-34:2	0.56
701.5592	*CerP, SM*	2.11
706.5381	*CerP, PC, PE*	0.66
716.5588	PE P-35:1	0.49
717.5905	*CerP, SM*	1.51
720.5901	*Cer, CerP, LPC, PE,*	2.15
723.5411	*SM*	2.31
728.5224	*CerP, PC, PE*	0.58
729.5252	*PG*	0.56
729.5905	*CerP, SM*	2.40
740.5200	PE 34:1	2.09
742.5721	*PC*	2.43
742.5745	*PC*	2.38
744.5513	*PC*	0.52
746.5694	PE 36:1	0.41
746.6057	*Cer, CerP, LPC, LPE*	1.79
747.6093	*HexCer*	1.79
748.5251	PE O-36:4	0.59
751.5724	*CerP, SM*	2.06
768.5877	*CerP, PC, PE*	2.93
768.5901	*PC*	2.85
770.6034	*LPC*	2.33
787.6687	*CerP, SM*	1.71
792.5877	*PC, PE*	3.76
792.5901	*PC*	3.77
794.5670	*PC, PE*	0.59
794.6034	*CerP, PC, PE*	3.19
796.6190	*CerP*	4.77
796.6214	*CerP*	4.72
798.5408	*CerP, PE*	0.59
818.6034	*CerP*	11.25

Cer – ceramides; CerP – ceramide phosphate; del – 1p/19q co-deleted samples; HexCer – hexosyl ceramides; LPC – lysophosphatidylcholines; LPE – lysophosphatidylethanolamines; m/z – mass to charge ratio; n-del- 1p/19q non co-delated samples; PC – phosphatidylcholines; PE – phosphatidylethanolamines; PG – phosphatidylglycerols; SM – sphingomyelins

Table S8. Patients characteristics.

No.	Type	Genetic Tests codeletion 1p/19q	Genetic Tests IDH1/2 mutation	Final Diagnosis*	Grade	2016 or 2021 WHO brain tumor classification	Age	Sex	Type of sampling
17	HGG	N	N	Glioblastoma, NOS	4	2016	73	M	Stored tissue
36	MEN	N	N	Meningioma	1	2021	53	F	Stored tissue
39	HGG	N	N	Glioblastoma, NOS	4	2016	72	F	Stored tissue
56	MEN	N	N	Meningioma	1	2021	80	M	Stored tissue
59	MEN	N	N	Meningioma	1	2021	62	F	Stored tissue
75	MEN	N	N	Meningioma	1	2021	55	F	Stored tissue
86	HGG	-	-	Glioblastoma, IDH-wildtype	4	2021	65	F	Stored tissue
87	HGG	-	-	Glioblastoma, IDH-wildtype	4	2021	67	F	Stored tissue
97	MEN	N	N	Meningioma	1	2021	44	F	On-site sampling
98	MEN	N	N	Meningioma	3	2021	72	F	On-site sampling
101	MEN	N	N	Meningioma	1	2021	59	F	On-site sampling
102	LGG	+	+	Oligodendroglioma, IDH-mutant, and 1p/19q codeleted	2	2021	33	F	On-site sampling
103	MEN	N	N	Meningioma	1	2021	55	F	On-site sampling
104	LGG	-	+	Astrocytoma, IDH-mutant	2	2021	32	M	On-site sampling
105	MEN	N	N	Meningioma	1	2021	41	F	On-site sampling
106	MEN	N	N	Meningioma	1	2021	70	F	On-site sampling
107	MEN	N	N	Meningioma	1	2021	58	M	On-site sampling
108	HGG	-	-	Glioblastoma, IDH-wildtype	4	2021	52	F	On-site sampling
109	MEN	N	N	Meningioma	1	2021	64	M	On-site sampling
110	HGG	-	-	Glioblastoma, IDH-wildtype	4	2021	64	M	On-site sampling
111	HGG	-	-	Glioblastoma, IDH-wildtype	4	2021	71	M	On-site sampling
112	LGG	-	+	Astrocytoma, IDH-mutant	2	2021	40	F	On-site sampling
114	MEN	N	N	Meningioma	1	2021	64	F	On-site sampling
115	MEN	N	N	Meningioma	1	2021	75	F	On-site sampling
117	HGG	+	+	Oligodendroglioma, IDH-mutant, and 1p/19q codeleted	3	2021	31	M	On-site sampling
118	MEN	N	N	Meningioma	1	2021	47	M	On-site sampling
119	MEN	N	N	Meningioma	1	2021	79	F	On-site sampling

120	HGG	-	-	Glioblastoma, IDH-wildtype	4	2021	50	M	On-site sampling
121	MEN	N	N	Meningioma	2	2021	42	M	On-site sampling
122	LGG	+	+	Oligodendroglioma, IDH-mutant, and 1p/19q codeleted	2	2021	52	F	On-site sampling
123	MEN	N	N	Meningioma	1	2021	42	F	On-site sampling
125	LGG	+	+	Oligodendroglioma, IDH-mutant, and 1p/19q codeleted	2	2021	59	M	On-site sampling
126	MEN	N	N	Meningioma	1	2021	51	F	On-site sampling
127	MEN	N	N	Meningioma	1	2021	66	F	On-site sampling
128	LGG	-	-	Pilocytic astrocytoma	1	2021	29	F	On-site sampling
129	HGG	-	+	Astrocytoma, IDH-mutan	4	2021	30	F	On-site sampling
130	HGG	-	-	Glioblastoma, IDH-wildtype	4	2021	69	M	On-site sampling
131	LGG	+	+	Oligodendroglioma, IDH-mutant, and 1p/19q codeleted	2	2021	71	F	On-site sampling
132	HGG	-	-	Posterior fossa ependymoma	3	2021	24	M	On-site sampling
133	HGG	+	+	Oligodendroglioma, IDH-mutant, and 1p/19q codeleted	3	2021	42	M	On-site sampling
134	MEN	N	N	Meningioma	1	2021	68	F	On-site sampling
135	HGG	-	-	Glioblastoma, IDH-wildtype	4	2021	78	F	On-site sampling
136	HGG	-	-	Glioblastoma, IDH-wildtype	4	2021	60	M	On-site sampling
137	MEN	N	N	Meningioma	1	2021	34	F	On-site sampling
138	HGG	+	+	Oligodendroglioma, IDH-mutant, and 1p/19q codeleted	3	2021	39	F	On-site sampling

***Tumor diagnoses were verified according to the 2021 WHO brain tumor classification. In some older cases before genetic testing was introduced into routine practice the 2016 WHO brain tumor classification was applied.**

F – female; M – male; MEN – meningioma; N – not known; NOS – not otherwise specified; HGG – high grade glioma; LGG – low grade glioma; "+" – present; "–" – absent
